# Expert opinions on the authenticity of moulage in simulation: a Delphi study

**DOI:** 10.1186/s41077-019-0103-z

**Published:** 2019-07-08

**Authors:** Jessica Stokes-Parish, Robbert Duvivier, Brian Jolly

**Affiliations:** 1 School of Medicine & Public Health, University of Newcastle, Newcastle, NSW Australia; 2 Department of Rural Health, University of Newcastle, Newcastle, NSW Australia; 3 Parnassia Psychiatric Institute, The Hague, The Netherlands

**Keywords:** Authenticity, Engagement, Instructional design, Instrument, Moulage, Simulation, Special effects makeup, SPFX

## Abstract

**Background:**

Moulage is a technique in which special effects makeup is used to create wounds and other effects in simulation to add context and create realism in an otherwise fabricated environment. The degree to which moulage is used in the simulated environment is varied; that is, there is no guide for how authentic it is required to be. To objectively assess whether a higher level of authenticity in moulage influences engagement and better outcomes, a common model to assess authenticity is required. The aim of this study was to explore expert opinions on moulage in simulation and develop an instrument for the classification of moulage in simulation.

**Methods:**

The instrument was developed in 3 phases: expert panellist recruitment, domain identification, and consensus rounds. A Delphi technique was used to explore themes of authenticity using Dieckmann’s Theory of Realism as a frame of reference. An initial list of elements was raised by a panel of international experts. The experts participated in a further four rounds of questioning, identifying and then ranking and/or rating elements of authenticity in moulage. A priori consensus threshold was set at 80%.

**Results:**

In round 1, 18 of 31 invited panellists participated, and a total of 10 completed round 5 (attrition 44%). As a result of the Delphi, the Moulage Authenticity Rating Scale was developed. Under the three domains of realism, 60 elements were identified by experts. A total of 13 elements reached the consensus threshold, whilst tensions regarding the necessity for authentic moulage were identified throughout the rounds.

**Conclusion:**

This study demonstrates the complexity of moulage in simulation, with particular challenges surrounding the experts’ views on authenticity. A prototype instrument for measuring moulage authenticity is presented in the form of the Moulage Authenticity Rating Scale (MARS) to further aid progress in understanding the role of authentic moulage in simulation.

**Electronic supplementary material:**

The online version of this article (10.1186/s41077-019-0103-z) contains supplementary material, which is available to authorized users.

## Background

Simulation is used in a wide range of disciplines, including healthcare, education, and other industry sectors (such as defence, mining, and engineering). It serves as an opportunity for practice where particular situations are not common or unsuitable/unsafe for practice [1, 2]. The success of simulation is largely dependent on instructional design and effective debrief techniques [3–5]. Key elements of instructional design include using appropriate fidelity to evoke realism [6]. Fidelity is thought to encompass physical, conceptual, and psychological components of simulation and is a topic of polarising discussion in the simulation community. Similarly, realism is suggested by Dieckmann (2007) as the degree to which a participant perceives reality in physical, semantical, and phenomenal aspects of reality [7]. Briefly, these modes of reality describe the actual physical components of reality such as the physical components of a manikin, and the semantical component of realism describes a conceptual kind of realism—for example, if bleeding occurs, a low blood pressure will result—and, finally, the phenomenal mode of realism. This kind of realism describes an emotional process, e.g. is the situation believable? [7]. The fiction contract is used to jump the hurdle of simulated realities, expecting that participants do the hard work of choosing to engage. The simulation community places importance on creating a level of realism, as noted in various literature [8, 9]. Encompassed in these broad aspects of ‘realism’ in simulation, are cues and set-up—thought to be essential for participant engagement [10, 11]. One kind of cue that contributes to physical realism is moulage.

The term moulage is used to describe special effects makeup (SPFX) and casting or moulding techniques that replicate illnesses or wounds [12]. Moulage has a long history in health professions education and anatomical teaching [13, 14]. Today, the techniques of moulage can include using make-up for bruising, creating wounds with wax, painting latex to achieve burns, and adding smells to the simulated environment. It can be costly to use these techniques, with specific training and equipment potentially required to effectively use moulage. It could be argued that moulage is only a physical component of realism; however, it may be also semantic and phenomenal. For example, moulage would presumably be a cue that assists a participant to move from A to B (semantical realism) and could aid in believability or emotional buy-in (phenomenal realism).

### What does the literature say about moulage?

Until recently there has been no attempt to explore the place of moulage in simulation other than accepting the status quo; evidence for moulage mainly focuses on instructional “how-to” guides and historical accounts in dermatology [14]. A recent systematic review explored the effectiveness of moulage on engagement in simulation, highlighting the paucity of credible research on moulage [12]. The research highlighted the disparities in use across disciplines, industries and simulation centres, identifying there was no evidence to support the need for moulage in simulation [12, 15]. Since the publication of this systematic review and after a moulage “call to arms” [14], Mills et al. (2018) demonstrated that the use of “highly realistic” moulage versus no moulage improved immersion in paramedicine students [16]. Novel research in the field of radiography identified students’ preferences for realistic moulage, with students commenting “… couldn’t tell it was makeup at first. Which I thnk [sic] is better as it looks more realistic …” [17]. Other research suggests that moulage improved realism; however, the quality of moulage was purportedly challenged by the lack of time to apply moulage [18].

**Fig. 1:**
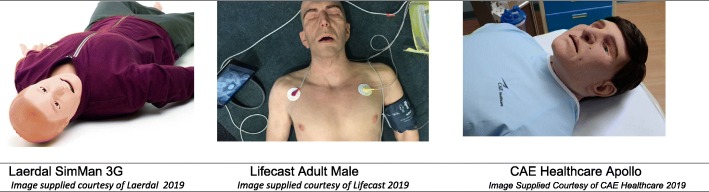
Comparison of manikin appearance

The term highly realistic, or realistic, is used frequently to create a distinction in simulation realism and in simulation manikin marketing [16, 19–21]. Despite this, there is no clear definition of what constitutes realistic. In fact, many educational outcomes papers that discuss the comparison of highly realistic or high-fidelity versus their low counterparts do not provide measures to define these classifications. For example, in Mills et al.’s (2018) study described earlier, there was no measurement to quantify “highly realistic”; however, in a study on burns training, Pywell et al. (2016) assessed the face validity of moulage in a comparison study for burns training [22]. In dermatology, authenticity of moulage rates highly due to the high-stakes conditions of diagnosing melanoma and other skin conditions [23–28]. In the field of gynaecology, researchers developed a rating tool to determine realistic simulator design [19, 22]. This is particularly interesting, given the inconsistency in industry approaches to designing simulators appearance—for example, Laerdal SimMan 3G versus LifeCast Adult Male Manikin versus CAE Healthcare Apollo (See Fig. 1). These manikins have varied levels of authenticity in their appearance—LifeCast, for example, employs SPFX teams to replicate the life-like appearance of manikins, including realistic skin and other details.

Some simulation designers and users argue that a high level of accuracy in the portrayal of moulage is necessary for the effectiveness of simulation; however, absolute authenticity is questioned in the current literature [9, 29, 30]. How does the use of moulage change the approach to instructional design of simulation? Before exploring its impact on instructional design, and then simulation participants, we posit that we must first understand what constitutes moulage authenticity. What is good moulage? And what, therefore, is bad moulage? How much time and money can one justify spending on moulage, when the cost-effectiveness of simulation is a priority for institutions and education [31]. We identified authenticity as a priority area for research in the use of moulage in health professions simulation [14], prior to exploring the many other possibilities in engagement, instructional design, and other areas of interest.

## Aims

This study aimed to develop an expert-generated instrument that defines moulage’s authenticity in simulation practice.

## Methods

The study was approved by the University of Newcastle Human Research Ethics Board (H-2016-0326).

Regarding the application of moulage, there are many techniques and moulage is used across multiple disciplines (within health, military, and other industry-based activities) across the world. For this research, we focused on healthcare simulation. To mitigate these challenges, a group consensus technique such as the Delphi method is capable of distilling expert opinion on key elements constituting moulage authenticity. The Delphi method is particularly flexible with achieving consensus through the use of electronic surveys to participants across the world, whilst maintaining anonymity [32]. In this section, we will outline the methodology employed to deliver the Delphi consensus method.

### Panellist selection

We sought to recruit a representative sample of experts with first-hand knowledge of moulage. An individual was classified as an expert if they worked with direct involvement in simulation instructional design and implementation (in particular, moulage), or as an experienced SPFX artist, involved in creating moulage or arts in anatomy. This wide-ranging sampling approach is supported by literature on Delphi technique to achieve high-quality outcomes [33, 34]—we chose to keep this broad due to the lack of evidence for moulage in the literature. Individuals were excluded from the study if they had no expertise in moulage/SPFX design or application, or, they had no expertise in the instructional design of simulation. The researchers screened participant responses for exclusion.

Panellists were identified via purposive sampling method to ensure adequate representation across geographic and educational variances (see Table 1). JSP approached simulation societies, journals and authors of papers that included moulage, and identified recommendations for expert participation, in addition to Google and LinkedIn searches, and via a snowballing technique informed by our previous Systematic Review [12] (Additional file 3). Literature does not provide clarity or consensus on the appropriate size of panels for a Delphi study; however, method experts recommend taking into account the number of experts in the subject area and the likelihood of completing the survey rounds [32]. In this study, nominees were individually contacted electronically, with requirements, study details and ethics approval outlined with a link participate in the first round. Each individual was allocated a unique identifier so they would remain anonymous to each other throughout the process, but identifiable to the researchers for administration purposes.

**Table 1:** Participant location and expertise

Geographic location of panellist	Expert panel roles	Expertise level (self-rated)	Highest degree held by expert	Frequency of moulage use
USA [7]	Moulage	Beginner (0)	No degree [2]	Daily [7]
Australia/New Zealand [6] Canada [3] UK [2]	Expert/technician [11]	Intermediate [10]	Certificate [2]	Weekly [8]
	Instructional designer [8]	Advanced [8]	Diploma [3]	Fortnightly [1]
	Simulation instructor [4]		Bachelor [4]	Monthly (0)
	Special effects expert [1]		Masters [6] PhD [1]	Occasionally [2]

The listed societies, editors, and authors assisted in the recommendation of panellists, resulting in 31 individuals being invited to participate. Eighteen responded to the invitation to participate (58%), with a total of 10 panellists remaining at the closing round (an attrition rate of 44%) (Table 2). Efforts were made to minimise attrition by clearly outlining the expected timeframes, priori consensus, and simple technology use [35].

**Table 2:** Panellist participation

Activity	Number
Invited to participate	31
Round 1	18
Round 2	15
Round 3	13
Round 4	12
Round 5	10

### Element generation

We used the typical Delphi method whereby the authors presented a series of open-ended questions to the experts to generate the initial elements [33]. The authors developed the round 1 questions using Dieckmann’s Theory of Realism as a conceptual framework [7]. In response to the need to understand moulage, we identified Dieckmann’s theory as an appropriate fit for developing a theoretical framework for moulage. This is because realism is contextual to both the environment and learning objectives, yet is subject to the participants’ judgement. Realism, in this theory, is composed of three domains—physical, semantic, and phenomenal. Physical realism refers to the actual physical representation, such as the characteristics of moulage, its textures, and colours; that is, how persuasive is the authenticity in your perception of reality [7, 14]. The semantical mode of realism refers to a conceptual type of realism, it is more about a participant’s relationship with the activity or story. That is, can s/he relate to the story? Is the representation plausible, could it occur in real life? In this instance, the moulage may not be authentic, but by way of the ‘fictional contract’ it is enough to help you regard it as authentic and predict what might happen next. For example, “if A occurred, B will happen”; Dieckmann et al. (2009) use the example of haemhorrhage—bleeding occurred; therefore, the blood pressure will decrease. *How* the information is shared is irrelevant, if interpretable information is shared. Finally, the phenomenal mode of realism is an emotional realism; engagement is reliant on your involvement in the situation and how persuasive it is to you, overall. Participants engage with the activity as if it were the real experience because they are emotionally engaged. For example, the moulage authenticity may be variable, but they engage with the narrative and situation because it makes sense to them.

This framework of realism creates the basis for starting our discussion on moulage in simulation, with attention to how moulage and engagement might interrelate. The use of Dieckmann’s Theory of Realism presents a novel approach to exploring moulage, operationalising an otherwise theoretical approach to realism in a practical way. Throughout this research, we framed our questions, interpretations, and analysis on this theory.

### Data collection tool

Data was collected using Survey Monkey, a secure, encrypted web-based electronic questionnaire system [36].

### Delphi procedure

**Fig. 2:**
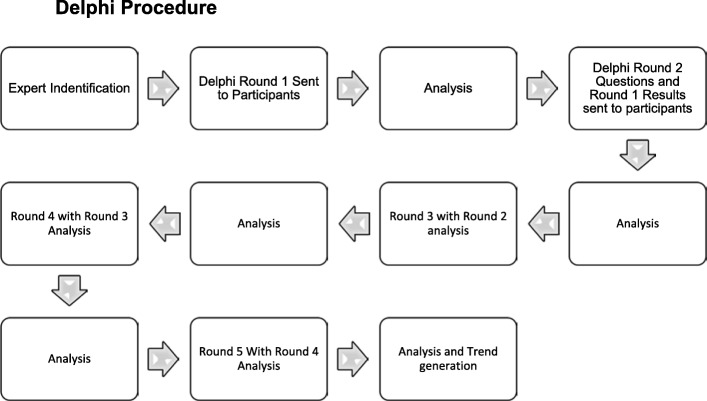
Delphi procedure

The survey was presented to the experts in multiple iterative rounds via the survey host (Survey Monkey) in late 2016. In the first round, designed to identify the basis for generating the elements of authentic moulage. Instructions for completing the Delphi and the surveys were shared with the panellists, followed by demographic questions regarding the participants, as well as a series of open-ended questions, and a request to list at least three elements that contribute to the appearance of moulage being real (Additional file 4). The questions were grouped into the three categories of Dieckmann’s realism—physical, semantic, and phenomenal. Statements regarding physical, semantic, and phenomenal realism were continually presented to the panel in subsequent rounds. In round 2, the elements identified in round 1 were thematically analysed by the researchers together (JSP, BJ) to group common themes. The themes were presented in round 2 in question format, seeking agreement via 5-point Likert scale (strongly agree to strongly disagree) as to whether the elements contribute to the appearance of moulage being real (e.g. “Colour is an element that contributes to the appearance of moulage being real”). Participants were requested to rank their top 5 elements out of the 14 physical elements. In round 3, providing feedback from round 2, the questions were re-framed to seek a rating of importance. Once the consensus threshold was reached on an element, it was removed from further rounds of questioning. The same process was repeated in round 4 and round 5. Total consensus on every element was not reached; therefore, the Delphi closed after the fifth round as per the priori consensus. In all rounds, experts were given the opportunity to add additional background, suggest revisions or additions via the use of free text boxes in the survey instrument. A flowchart of the process followed can be seen in Fig. 2.

### Priori consensus

In line with recommendations for Delphi technique methodology, a priori consensus was set at 80% or five rounds of surveys, whichever was sooner. The data was analysed using Microsoft Excel (Version 15.33). The results were shared in each round with the respective question.

## Results

### Initial elements generated

In round one, a total of 60 elements were raised as contributing to the authenticity of moulage (Additional file 1). Of the 60 elements presented by the experts in relation to the appearance of moulage, the most common element of physical authenticity was “colour” (*n* = 11). Coming in at second, was likeness to real world. The elements generated were grouped into common themes, resulting in the population of 17 elements generated in round 1 (see Table 3).

**Table 3:** Common elements in round 1

Common elements generated in round 1 (*n* = number of times listed)	
Colour (11)	
Likeness to real world (7)	
Texture (6)	
Position (4)	
Smell (4)	
Blend (3)	
Depth (3)	
Feel (3)	
In conjunction with setting (3)	
Size (2)	
Shape (2)	
Relevant/logical (2)	
Not over done (2)	
Consistency (1)	
Scale (1)	
Detail (1)	
Sound (1)	

In round 2, panellists were asked to rate their agreement with the statements presented and rank their top five (5) elements of physical authenticity. To calculate the mean rank of items, the sum of answers was divided by the number of panellists, minus the missing data. Likeness to real world was overwhelmingly highest ranked, with 87.5% of respondents ranked this element in the top 5. It was followed closely by “anatomical correctness” (62.5% ranked in the top 5), “position” (50%), “colour” (62.5%), and “detail” (68.7%) (see Table 4). Some items scored very closely or within the mean ranking numbers listed below, such as “scale” and “texture”. However, we analysed that these had significantly higher missed answers and therefore less votes, meaning that the weight of the response was lower.

**Table 4:** Top five elements by mean ranking

Element	Number of respondents who ranked item (%)	Mean ranking
Likeness to real world	14/16 (87.5%)	1.43
Anatomical correctness	10/16 (62.5%)	2.30
Position	8/16 (50%)	2.50
Detail	11/16 (68.7%)	3.09
Colour	10/16 (62.5%)	3.70

In addition to the listing of elements contributing to physical authenticity, panellists were asked to comment on what level of authenticity is required for the moulage to be conceptually believable (semantic realism) and how moulage contributes to choices (phenomenal realism). Seventeen elements presented were in relation to semantic realism, and 16 elements were in relation to phenomenal realism. After combining common elements, there were 15 and 9 resulting elements, respectively.

### Subsequent rounds

In round 3, we reframed the questions to rate the level of importance of each element to better understand the consensus view. The elements that reached consensus early were clear: “likeness to real world”, “anatomically correct”, “position”, and “detail”. Table 5 provides a full summary of the results of each element.

**Table 5:** Results of rounds 3–5

Element	R3 (%)	R4 (%)	R5 (%)
Colour	77	83 threshold met	–
Size	23	66	100 threshold met
Consistency	31	50	68
Position	85 threshold met	–	–
Depth	15	17	55
Shape	49	66	100 threshold met
The feel	62	67	44
Smell	46	41	33
Scale	54	59	67
Texture	31	59	55
Detail	85 threshold met	–	–
Sound	23	25	33
Likeness to real world	100 threshold met	–	–
Anatomically correct	93 threshold met	–	–
The moulage fits logically within the scenario	100 threshold met	–	–
The moulage is presented as a part of props/scene	84 threshold met	–	–
The moulage is at a sufficient level so as not to distract/confuse the participant	92 threshold met	–	–
Good facilitation can mitigate low realism	69	75	77
Simulation orientation can mitigate low realism	61	66	89 threshold met
The moulage is well-timed, where appropriate	92 threshold met	–	–
The moulage fits with the scenario	100 threshold met	–	–
The moulage makes use of all senses	76	66	78

Throughout the rounds of questioning, the experts were invited to provide further comments to explain their answers or add to the discussion. With little theoretical underpinnings for moulage in simulation, the discussion amongst the experts contributed new ideas and concepts to the approach to the use of moulage. Early on the panellists identified the priority for realistic moulage. Examples of responses include

“the more realistic a situation or environment is, the more believable it will be for the learner and the easier it will be to engage”. (Participant 15: Intermediate Moulage Expertise, USA).

“There definitely needs to be a relatively high level of authenticity so that the learners ‘buy in’ to the scenario. If the learners deem the scenario unrealistic, they will not engage fully, and get nearly as much out of it, as they would if it was more realistic.” (Participant 2: Intermediate Moulage Expertise, Australia).

“… Obviously the higher the level of realism the more engagement of the learner ….” (Participant 3: Advanced Moulage Expertise, USA).

In describing the necessity for authenticity, experts identified that realism is learner dependent:

“… For example a red cloth could symbolise blood in a scenario if the learner is willing to believe that the red cloth is to be interpreted as blood. …” (Participant 18: Advanced Moulage Expertise, USA).

“Some novice students can engage in low fidelity simulations as they are not yet equipped to process information under pressure. For the more experienced, scenarios resembling reality as closely as possible are advantageous.” (Participant 16: Intermediate Moulage Expertise, Australia).

The thoughts from the experts regarding the necessity of authenticity presented some interesting tensions. Firstly, they felt that the accuracy of moulage directly relates to the relevance of the scenario, contributing to diagnoses and behaviours. Secondly, they felt that moulage that was not authentically portrayed can be confusing.

“the visual effect must be similar to the lived experience as this allows the student to connect the dots and transfer concepts and theory to practice”, (Participant 4: Intermediate Moulage Expertise, Canada).

“Bad or excessive moulage can be worse than none as it can remove the focus away from learning to ‘gazing’ at the theatrical aspects as an independent entity. Can be used by some learners as an excuse for poor performance - ‘it was naff and not anything like real life so I couldn’t apply myself like real life’”, and (Participant 7: Intermediate Moulage Expertise, Australia).

“The more realistic looking and feeling the moulage allows trainees to become more invested” (Participant 13: Advanced Moulage Expertise, Australia). However, the issue of cost and time became a central point of discussion—some experts described the use of cue cards (with written cues) instead of moulage. The final instrument can be viewed as Additional file 2.

## Discussion

We performed an international Delphi study that led to 13 indicators for moulage authenticity. To our knowledge, this is the first detailed expert identification of indicators of moulage authenticity.

Clear agreement on elements such as colour, likeness to real world was different to size and shape, for example. In these instances of meaning making with “size” and “shape”, the element initially appeared to have no agreement and in later rounds reached agreement. The reason for this is not entirely clear, but could be due to the attrition in the rounds. That is, perhaps the experts who had a view of disagreement dropped out of the study; therefore, the resulting “consensus” may not be representative of the initial group. Another point of view might be that the areas of clear consensus (likeness to real world, colour, etc.) were identified very early on, and perhaps these other elements were areas of “emerging” consensus. That is, now that the clear winners were removed, the grey areas could be adequately considered by the experts. We argue that there is confidence in the answers due to the ranking process implemented in round 2. The top five (via mean ranking) clearly correlate with the items reaching consensus in the latter rounds.

On exploring the importance of the top five elements, we were unsurprised to see “likeness to real world” as the area of priority. The consensus on “appearance”, “feel”, and “anatomical accuracy” are consistent with research on simulator realism [19]. Interestingly, one might argue that likeness to real world is a proxy term for authenticity. If so, the implication is not insignificant. If likeness to real world is rated as the single most important element of moulage, does this then mean that authentic portrayal of moulage is necessary, hands down? Considering Dieckmann’s realism, physical, semantic and phenomenal the element of authenticity directly relates to the physical aspect of realism [7]. However, it stands to reason that there is a direct relationship between authentic physical portrayal and the semantic and phenomenal aspects of realism. A more authentic portrayal of the semantical element might enable an easier leap from “A to B” and a more emotional “buy-in” for the phenomenal realism. Realistic moulage improves immersion (phenomenal realism), but may negatively affect clinical performance; however, in this research there is no measurement of the realistic portrayal of moulage [16]. Moulage rates higher in face validity when applied by a trained make-up artist versus a simulation technician, but did not appear to hinder performance [22]. In our conceptual work, we suggested that perhaps moulage could be a conceptual representation of reality; however, the use of the term likeness to real world and its rating of importance might suggest otherwise [14]. Hamstra et al. argue that simulators should be assessed on how closely they resemble real life (physical resemblance) and how closely the simulator functions like a real human would (functional task alignment) [30]. If applied to moulage authenticity, then the likeness to real-world aspect is relevant to the purpose at hand. For example, if a trauma scenario is presented with the purpose of training undergraduate medical students how to conduct primary and secondary assessments, whilst maintaining situational awareness, we would argue likeness to real world is relevant for the purpose of learning. If moulage is not authentic, the learner might dismiss the relevance or assume it is not important.

**Fig. 3:**
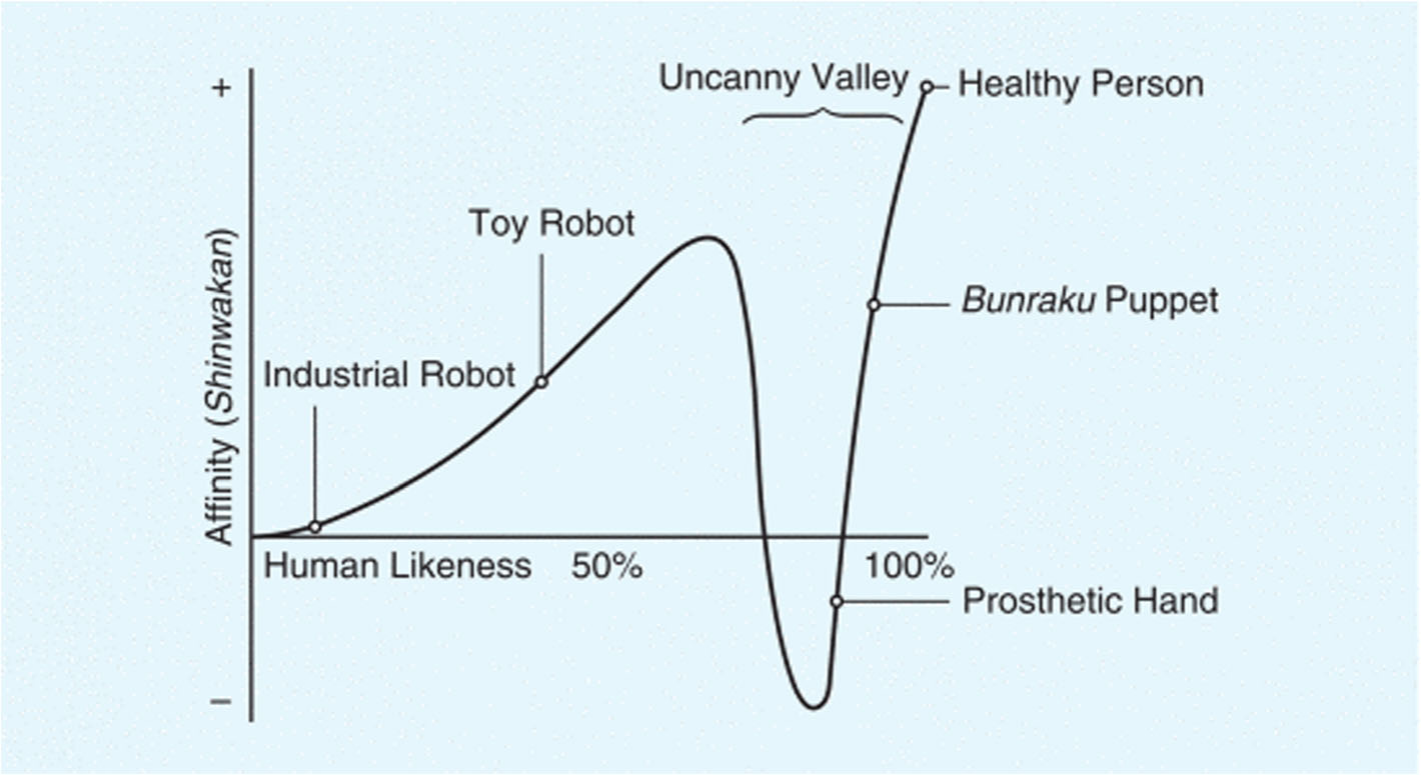
The Uncanny Valley (Mori, 1970)

Another perspective when considering the authenticity of moulage is the Theory of the Uncanny Valley (Fig. 3), whereby the pursuit of authenticity in robotics and avatars created a sense of fear instead of engagement—it looks familiar, yet it is simultaneously strange [37, 38]. This theory proved that the inconsistency in the realism portrayed caused individuals to have internally conflicted opinions about perceptual persuasiveness. If applied to authentic moulage, perhaps authenticity is relative to the surrounding environment. For example, the moulage might be more “strange” when applied to a mannequin, as compared to a Simulated Patient (a real person trained to portray a particular illness or effect) [39].

A discussion regarding authentic moulage is timely. How would a framework for moulage authenticity apply to our practice? Firstly, we suggest that understanding moulage with a theoretical lens would add weight to the importance of its application in simulation. Sentinel work on simulation foundations either identifies moulage as a side note or does not included it in the discussion of realism at all [8]. Secondly, by using this instrument, simulation might be assessed in more measurable ways, enabling designers to make conclusive decisions about the application of moulage in their simulation design. An extension of this might include its use as a marker of authenticity when using simulation as a form of assessment, potentially improving the validity and transferability of the simulation-based assessment. Additionally, we suggest that the authenticity rating could be applied to the comparison of manikins and their appearance, or in comparison of manikins and simulated patients, further extending our understanding of the use of manikins and simulated patients.

### Limitations

The research did not include experts from South America, Africa, or Asia. We are unsure if this was due to the methods employed or the patterns of research in those continents. Purposive sampling was selected as the method due to the increased likelihood that panellists selected possessed the necessary expertise. Perhaps this reflects the level of maturity of simulation in these continents, whereby research centres, journals, and attendance is not as predominant as in western societies. Additionally, it is worth considering the bias that might be inadvertently demonstrated by publishing bodies—it is known that the majority of authors in high-ranking medical education journals, for example, are from 5% of the world [40, 41].

Despite planned administrative processes and clear objectives, the administration of this Delphi was time-consuming [42]. This led to a delay in some of the survey deployments, which may have contributed to the attrition rate. Despite this, the attrition rate is not too dissimilar to other Delphi work—the larger the panel, the greater the attrition [43]. Some of the attrition may be attributed to a lack of interest or expertise (for example, the SPFX artist dropped out after round 1).

Additionally, the lack of information regarding moulage may have contributed to the need for the full five rounds of questioning and the lack of consensus. Perhaps the paucity of research around moulage and the absence of a theoretical framework contribute to the contradictory views and the lack of consensus on some issues. We would argue that, similar to Mullen (2003), a multiple “correct” answers are better than a “unanimously agreed wrong answer” [43].

As for other limitations, we do not present findings of a content validity study in this paper. This work is not included as we felt it would detract from the discussions raised by the experts and falls outside the scope of this study.

### Future directions and conclusion

The potential for authenticity and moulage remains largely unexplored. Experts on moulage present thoughtful ideas as to how moulage might contribute to realism in simulation and what authentic moulage might mean in this context. The development of this instrument presents an opportunity to measure the impact of authentic moulage on various aspects in simulation. To verify the instrument’s representativeness, content validity should be assessed by means of an expert survey. Additionally, there is a need to assess the reliability could be verified in a series of trials, whereby moulage elements with a range of authentic and inauthentic features would be rated for authenticity by simulation experts, clinicians, and students. Additionally, the fit of this instrument across other domains, such as augmented, virtual, or mixed realities, could benefit the wider simulation community.

Further areas of exploration might include the comparison of junior versus senior learners, exploring how the approach to simulation context and cues differs. This could be done applying the moulage instrument to create clear distinction between high and low authentic moulage.

This paper presents novel work in the field of both authenticity and moulage. Tensions remain present in regard to the necessity of moulage, how it is applied and the level of authenticity required to be portrayed in a variety of settings. There is a clear imperative to explore authentic moulage further to benefit the simulation community.

## Additional files

Additional file 1: Elements identified in round 1 of Delphi. (DOCX 99 kb)

Additional file 2: Moulage authenticity rating scale (MARS). (DOCX 64 kb)

Additional file 3 Round 1 Survey Content. (DOCX 22 kb)

Additional file 4: Recruitment. (DOCX 55 kb)

## Data Availability

The datasets used and/or analysed during the current study are available from the corresponding author on reasonable request. Initial data generated or analysed during this study are included in this published article—“Additional file 1”.
