# Supplementary material for: Expert opinions on the authenticity of moulage in simulation: a Delphi study
Source: Adv Simul (Lond). 2019 Jul 8;4:16. doi: 10.1186/s41077-019-0103-z (PMC6615296; doi:10.1186/s41077-019-0103-z)
Supplement: Supplementary file 1 — Elements identified in round 1 of Delphi. (DOCX 99 kb) [file 41077_2019_103_MOESM1_ESM.docx]

**Additional File 1 – Elements identified in Round 1 of Delphi**

1. My specialty is wounds so they must look like the wounds seen in practice,
2. they must mimic characteristics and
3. blend into the skin or manikin
4. Colour
5. Size
6. Consistency
7. Position
8. Appropriate relation to the regional anatomy and physiology. The correct size of bone for the injured area for example.
9. Depth and shape. A three dimensional wound or area of involvement presents a more realistic cue to the student especially if the objectives of the simulation involve analysis of diagnostics in order to develop a plan of care. A raised mole or an infected wound with tunneling for example.
10. The moulage is logical for the case.
11. In other words not moulage done just for the sake of doing it or a wow factor.
12. Looks like,
13. smells like and
14. feels like the real thing
15. scale,
16. colour,
17. texture
18. Texture/Depth,
19. Color and
20. Detail.
21. Likeness of bodily fluids to real fluids especially blood;
22. match of items to story presented;
23. presented as part of other props/scene setting
24. The dirt, blood, pus of any drainage adds to the realism There is always some redness to the areas involve from slight to more intense
25. Coloring of the wound, sore scare e.c.t needs to needs to be as close as possible
26. The application of a wound, scar, sore edges need to be flush with the surface in order to look like part of the skin
27. texture
28. colour
29. smell
30. Interactive training experience that mimics realism.
31. Sensory utilization
32. Simple to defined wound development
33. Knowing what you are creating on a mannequin or person looks like in real life. It is vital to visualize from pictures or an actual wound what it is you are trying to replicate.
34. Less is more. Starting small is a key element when creating any type of wound. Over doing your work takes it away from the realistic to a science fiction element.
35. Using the right materials. Matching skin color and what a real live wound looks like is majorly important to develop realism for the learning. Example, high shadow (blue) can provide the depth to a bruise but if it is frosted and creates a glimmer in the light, that's not real. In the end it takes PRACTICE before using any material in a simulation.
36. Colour
37. Shape
38. Texture
39. Use of products that are realistic looking (ie skin colour and wound colour matching)
40. Use of products that are realistic feeling.
41. Design of moulage that is based on clinical fact (ie use clinical photographs).
42. Color,
43. texture, and
44. positioning all contribute to the realism of moulage and increase the fidelity of the simulation.
45. Environment,
46. texture, context,
47. sound,
48. smell,
49. feel
50. Matches the learners expectation of realism
51. Blends in seamlessly with the narrative/story
52. Not obviously make-up
53. Knowledge of real wounds then duplicate them.
54. The amount of blood
55. the textures colors and
56. thickness of wounds and
57. thickness of blood and smells
58. Appropriate color,
59. size and
60. placement
