# Supplementary material for: Expert opinions on the authenticity of moulage in simulation: a Delphi study
Source: Adv Simul (Lond). 2019 Jul 8;4:16. doi: 10.1186/s41077-019-0103-z (PMC6615296; doi:10.1186/s41077-019-0103-z)
Supplement: Supplementary file 2 — Moulage authenticity rating scale (MARS). (DOCX 64 kb) [file 41077_2019_103_MOESM2_ESM.docx]

| Element | Low Authenticity 0 | Mid Authenticity 1 | High Authenticity 1 |
| --- | --- | --- | --- |
| Colour |  |  |  |
| Size |  |  |  |
| Position |  |  |  |
| Shape |  |  |  |
| Detail |  |  |  |
| Likeness to real world |  |  |  |
| Anatomically correct |  |  |  |
| The moulage fits **logically** within the scenario |  |  |  |
| The moulage is presented as a **part of props/scene** |  |  |  |
| The moulage is at a **sufficient level** so as not to distract/confuse the participant |  |  |  |
| **Simulation orientation** can mitigate low realism |  |  |  |
| The moulage is **well-timed**, where appropriate |  |  |  |
| The moulage **fits** with the scenario |  |  |  |
| **COLUMN TOTAL** |  |  |  |
|  |  | **TOTAL SCORE** |  |

**Scoring Instructions**: Rate each element according to the scale (0 – low, 1 – mid, 2 – high). The higher the score, the higher the authenticity.

Authored by: Jessica Stokes-Parish, Robbert Duvivier, Brian Jolly
