# Supplementary material for: Expert opinions on the authenticity of moulage in simulation: a Delphi study
Source: Adv Simul (Lond). 2019 Jul 8;4:16. doi: 10.1186/s41077-019-0103-z (PMC6615296; doi:10.1186/s41077-019-0103-z)
Supplement: Supplementary file 3 — Round 1 Survey Content. (DOCX 22 kb) [file 41077_2019_103_MOESM3_ESM.docx]

Additional file 3

*Survey Content*

**Round 1**

INFORMATION

Thank you for agreeing to participate in this Delphi survey on moulage authenticity.

This questionnaire round is the first of up to five rounds of the survey. You will have the opportunity to revise your answers with subsequent rounds of the survey.

In these surveys, you will be asked to develop priorities among the authenticity of moulage. Most of the questions can be answered with only a single selection. Where appropriate, a space is also provided for you to comment on the underlying reasons for your responses.

•

Once we have received responses from all panellists, we will collate and summarise the findings and formulate the second questionnaire. You should receive this in the next month.

We assure you that your participation in the survey and your individual responses will be strictly confidential to the research team and will not be divulged to any outside party, including other panellists.

QUESTIONS

Please complete the form below. Your details will be kept strictly confidential.

PAGE 1 – Sampling

1. I have read the explanatory statement and consent to participation in this research.
   1. Yes
   2. No
2. Please state your name
3. Phone number
4. Email address
5. Role title
6. Work address
7. Qualifications
   1. PhD
   2. Masters
   3. Bachelor
   4. Certificate
   5. No qualification
8. Please enter your country that you are currently living in:
9. What would you describe as your area of expertise in relation to simulation (Eg. Technician, special effects/moulage expert, instructional design)
10. Are you actively practicing moulage on a regular basis
    1. Yes
    2. No
11. How would you describe your level of expertise with moulage (or SPFX):
    1. Beginner
    2. Intermediate
    3. Advanced
12. Are you regarded as an expert in your field of practice
    1. yes
    2. no
13. Please provide detail on your response to question 12 (eg. Publications, workshops delivered, examples of moulage work):
14. Which continent do you live in:
    1. North America
    2. Australia
    3. Europe
    4. Asia
    5. Africa
    6. Other:

PAGE 2 – Domains of authenticity

Moulage is known in the simulation environment as the use of special effects makeup techniques or casting to add cues or elements of reality to a simulation. Examples of moulage might include applying bruises or illness effects through the use of makeup, or the use of pre-made wounds on a manikin or person.

The following questions are about what elements you feel contributes to realistic moulage. Please answer in as much detail as possible.

1. List at least 3 elements that contribute to the appearance of moulage being real (that is, what physical aspects of the moulage make it look real?)
2. Please list a brief explanation (2 or 3 sentences) of the importance of each factor that you listed in the previous question

Realism is a context discussed in simulation literature frequently. The level of realism to facilitate learning is relative to the learning objectives of the activity. What we are exploring here is how realism influences engagement in the scenario. Realism encompasses statements such as "The scenario was highly realistic" and refers to how the simulated situation reflects real life.

To explore realism and engagement further, we use the framework of realism presented by Dieckmann et al (2009) in which the authors discuss “physical”, “semantical” and “phenomenal” modes of realism.

Physical refers to the actual physical representation, such as the characteristics of the moulage, it’s textures and colours (eg. how persuasive is it in your perception of reality).

(Ref: Dieckmann, P., D. Gaba, and M. Rall, Deepening the theoretical foundations of patient simulation as social practice. Simulation In Healthcare: Journal Of The Society For Simulation In Healthcare, 2007. 2(3): p. 183-193.)

1. How can the physical appearance of moulage in a simulation influence (positive or negative) a learner’s engagement?
2. Is there anything more you wish to add?

The semantical mode refers to a conceptual type of realism. It is more about your relationship with the activity or story – can you involve yourself in the story? What would typically happen? Is it plausible that the narrative or story presented to you would occur? In this instance the moulage may not be authentic, but by way of the ‘fictional contract’ it is enough to help you theorise what might happen next. For example, if "A occurred, B will happen"; Dieckmann et al (2009) use the example of haemhorrhage - bleeding occurred, therefore the blood pressure will decrease. How the information is shared is irrelevant, as long as interpretable information is shared.

1. What level of realism (authenticity) is needed to engage with a scenario for it to be considered conceptually believable?
2. Is there anything more you wish to add?

The phenomenal mode is an emotional type of realism, where engagement is reliant on your involvement in the situation and how persuasive it is to you. You engage with the activity as if it were the real experience because you are emotionally engaged. For example, the moulage authenticity may be variable, but you engage with the narrative and situation because it makes sense to you.

1. How might the appearance of moulage influence (positive or negative) the clinical choices the learner makes about a scenario?
2. How might the appearance of moulage evoke an emotional response?
3. Is there anything more you wish to add?

Thank you for participating in Round 1 of the survey.
