# Supplementary material for: Expert opinions on the authenticity of moulage in simulation: a Delphi study
Source: Adv Simul (Lond). 2019 Jul 8;4:16. doi: 10.1186/s41077-019-0103-z (PMC6615296; doi:10.1186/s41077-019-0103-z)
Supplement: Supplementary file 4 — Recruitment. (DOCX 55 kb) [file 41077_2019_103_MOESM4_ESM.docx]

Recruitment

| **Society** | **Journal** |
| --- | --- |
| Australian Society for Simulation in Healthcare/Simulation Australasia (ASSH) | Society for Simulation in Healthcare Journal |
| Society for Simulation Applied to Medicine (SESAM) | Advances in Simulation |
| Asia Pacific Simulation Alliance | BMJ STEL |
| Society for Simulation in Healthcare (SSH) Chair SOTS |  |
| Pan Asia Society for Simulation in Healthcare |  |
| ASIASIM.ORG |  |
| The Gathering of Healthcare Simulation Technology Specialists (SimGHOSTS) |  |
| Association for Simulated Practice in Healthcare (ASPiH) |  |
| Association of Standardized Patient Educators (ASPE) |  |
| SIM-One |  |
| New Zealand Association for Simulation in Healthcare (NZASH) |  |
|  |  |
|  |  |
|  |  |
|  |  |
|  |  |
